# Neural responses to social decision-making in suicide attempters with mental disorders

**DOI:** 10.1186/s12888-022-04422-z

**Published:** 2023-01-09

**Authors:** Shuang Liu, Moxin Duan, Yiwei Sun, Lingling Wang, Li An, Dong Ming

**Affiliations:** 1 Academy of Medical Engineering and Translational Medicine, Tianjin University, Tianjin, 300072 China; 2 School of Education, Tianjin University, Tianjin, China; 3 School of Precision Instruments and Optoelectronics Engineering, Tianjin University, Tianjin, China

**Keywords:** Decision-making, Suicide attempt, EEG, ERP, Theta-gamma PAC, Beta-gamma PAC, Ultimatum game

## Abstract

**Background:**

Decision-making deficits have been reported in suicide attempters and may be a neuropsychological trait of vulnerability to suicidal behavior. However, little is known about how neural activity is altered in decision-making. This study aimed to investigate the neural responses in suicide attempters with mental disorders during social decision-making. Electroencephalography (EEG) were recorded from 52 patients with mental disorders with past suicide attempts (SAs = 26) and without past suicide attempts (NSAs = 26), as well as from 22 age- and sex- matched healthy controls (HCs) during the Ultimatum Game (UG), which is a typical paradigm to investigate the responses to fair and unfair decision-making.

**Methods:**

MINI 5.0 interview and self report questionnaire were used to make mental diagnosis and suicide behavior assessment for individuals. Event-related potential (ERP) and phase-amplitude coupling (PAC) were extracted to quantify the neural activity. Furthermore, Spearman correlation and logistic regression analyses were conducted to identify the risk factors of suicidal behavior.

**Results:**

ERP analysis demonstrated that SA patients had decreased P2 amplitude and prolonged P2 latency when receiving unfair offers. Moreover, SA patients exhibited greater negative-going feedback-related negativity (FRN) to unfair offers compared to fair ones, whereas such a phenomenon was absent in NSA and HC groups. These results revealed that SA patients had a stronger fairness principle and a disregard toward the cost of punishment in social decision-making. Furthermore, theta-gamma and beta-gamma PAC were involved in decision-making, with compromised neural coordination in the frontal, central, and temporal regions in SA patients, suggesting cognitive dysfunction during social interaction. Statistically significant variables were used in logistic regression analysis. The area under receiver operating characteristic curve in the logistic regression model was 0.91 for SA/HC and 0.84 for SA/NSA.

**Conclusions:**

Our findings emphasize that suicide attempts in patients with mental disorders are associated with abnormal decision-making. P2, theta-gamma PAC, and beta-gamma PAC may be neuro-electrophysiological biomarkers associated with decision-making. These results provide neurophysiological signatures of suicidal behavior.

## Background

Suicide is a leading cause of death worldwide. An estimated 700,000 individuals die by suicide worldwide annually, which corresponds to a suicide rate of 11.4 per 100,000 people. However, our ability to understand, predict, and prevent suicide remains inadequate [1, 2]. Previous research has shown that past suicide attempts are the best predictors of a future suicide attempt [3]. A better understanding of the risk factors of suicide, particularly in suicide attempters, is needed to improve suicide prevention programs.

Neurocognitive attributes, such as decision-making, mediate thought-behavior relationships and may predispose individuals with suicide ideation to act on their thoughts and attempt suicide [4]. Suicide is an extreme decision-making behavior that is often closely related to a social crisis. Previous studies have shown that the social decision-making of suicide groups is defective and over-sensitive to social events (such as unfair treatment), which may promote a suicide attempt [5–11]. The socioeconomic Ultimatum Game (UG) has been proposed as a tool to observe the influence of perceived social injustice on social interaction decision-making [12, 13]. In the UG, participants are required to accept or reject monetary offers from an anonymous participant. Despite clear information that the goal of the game is the same for all participants, i.e., to gain the maximum amount of money, participants often react differently [14, 15]. Szanto et al. observed that high-lethality suicide attempters rejected unfair offers based primarily on the perception of inequity, but disregarded their economic loss [16]. A later study found a higher likelihood of rejecting offers in the acutely suicidal group compared to healthy controls [17]. These observations suggest that individuals prone to suicide show an increased sensitivity to social injustice. Suicide attempters tend to make adverse decisions and show impaired social functioning. In the future, additional research is needed to explore the characteristics of fairness in social decision-making performance, as well as the underlying neural mechanisms, in suicide attempters.

A deficit in decision-making may be the result of impaired cognitive functions. Decision-making processes occur in the timeframe of several hundred milliseconds. Thus, they are best studied using high temporal resolution methods, such as electroencephalography (EEG), to investigate rapid changes in cognitive processing and evaluate the reasons behind suicide attempts.

Event-related potentials (ERPs) are useful for studying decision-making. Its high temporal resolution identifies potential psychological activity and cognitive processes during decision-making. In the UG game, early decision-making processes involve attention-related perceptual processing, reflected by a negative component of about 100 ms (N1). A positive component of about 200 ms (P2) was associated with integration of motivational information, specifically reward-related information from the mesencephalic dopamine system, from an ongoing event [18–21]. In addition, several studies suggested the presence of a major ERP component located in the fronto-central region within 250–350 ms after stimulus onset [22–24]. This component, called the feedback-related negativity (FRN) component (also referred to as the medial frontal negativity), was more pronounced following negative outcomes compared to positive outcomes and was sensitive to the degree of deviation of an outcome from expectations [25]. The FRN amplitude was more pronounced in response to unfair offers than to fair offers [26, 27]. However, the aforementioned characteristics of suicide attempters during the UG are unclear and need further exploration.

Interactions between oscillations of different frequencies are termed as phase-amplitude coupling (PAC). PAC has received significant attention in the last decade due to its potential relevance for evaluating healthy and pathological brain functions [28–34]. Several studies have reported that different neurological diseases can alter PAC characteristics for rhythmic activity [35–39]. Therefore, exploring PAC can yield novel insights into neurocognitive processes.

The best known example of PAC was demonstrated in the human neocortex [40], where the theta rhythm phase was found to modulate the power of gamma oscillations. However, subsequent studies showed that PAC is neither limited to theta-gamma coupling. In particular, in the frontal, posterior, and parietal human cortices, PAC has also been reported during auditory, visual, linguistic, and memory tasks [41–43]. Other PAC combinations of low- and high-frequency rhythms have also been detected: alpha-gamma and beta-gamma [44–46]. Recent reports have suggested that theta-gamma PAC (TGC) in the rat orbitofrontal cortex discriminates between correct and incorrect decisions during associative learning. The results suggested that higher TGC is associated with good decisions [47]. Moreover, abnormal beta-gamma PAC activities have been identified in recordings from the scalp of patients with Parkinson’s disease, reflecting cognitive impairment [48]. It will be interesting to see whether PAC is correlated with cognitive symptoms in suicide attempters. This might increase the potential application of PAC detection and modulation for the diagnosis or treatment of neurological diseases. However, it is unclear whether and how PAC is involved in suicide attempts during social decision-making.

The primary goal of the present study was to clarify the neural basis of unfairness in conditions of social decision-making in suicide attempters with mental disorders in the UG game. We hypothesized that, compared to non-suicide attempters (NSAs) and healthy controls (HCs), suicide attempters (SAs) would show abnormal decision-making in response to unfairness, due to potential oversensitivity to unfairness. We also hypothesized that SA might have altered PAC during social decision-making, accoding to decision-making deficits were reported in SA. Furthermore, most people who think about suicide would never attempt suicide, we hypothesized that distinctive alterations in brain activation during decision-making might differentiate SAs from NSAs. These analyses have the potential to shed new light on the performance, and the underlying neural mechanism, of SAs during social decision-making to further understand the pathophysiological mechanism of impaired social function in SAs.

## Methods

### Participants

Fifty-two patients with mental disorders were recruited from the Tianjin Anding Hospital in Tianjin, China. Among them, there were 26 SAs with a lifetime history of suicide attempts and 25 NSAs with no lifetime history. Exclusion criteria for both groups were 1) history of diagnosed with brain organic diseases or chronic somatic diseases; 2) color blindness and color weakness; 3) an inability to read and understand the materials given to them; and 4) unclear SA history. A Chinese version MINI 5.0, which was shown to have good reliability and validity [49], was used in our study to screen psychiatric disorders and history of suicide attempts. It was mainly used to screen and diagnose 16 axis I mental disorders and one personality disorder in the Diagnostic and Statistical Manual of Mental Disorders, Fourth Edition (DSM-IV) and the International Statistical Classification of Mental Disorders (ICD-10).

We also included 22 HCs matched to the individuals with mental disorders in terms of age and sex in the communities, streets, schools, and other locations in Tianjin, China. The exclusion criteria of the HC group were 1) history of sustained head injury or other neurological or psychiatric disorders. 2) color blindness and color weakness; 3) an inability to read and understand the materials given to them. Psychiatric disorders were also excluded using MINI.

### Clinical assessment

Beck’s Depression Inventory (BDI-13) was used to determine the severity of depressive symptoms and the Cronbach’s coefficient was 0.94 in this study. Higher total scores corresponded to more severe depression [50]. Suicidal ideation was assessed using the Beck Scale for Suicide Ideation (BSSI) [51]. In this study, we used the Chinese version of BSSI, which contains 19 items and the Cronbach’s coefficient of BSSI is 0.94. The State Trait Anxiety Inventory (STAI) was developed by Spielberger et al. Our study used the Trait Anxiety Inventory (T-AI), including 20 items to evaluate recurrent anxiety. The Cronbach’s coefficient of the STAI is 0.93 in our study [52].

The clinical and socio-demographic data of patients and controls are summarized in Table 1. All participants were aged 18–65 years, without speech comprehension disorder or color blindness, and with a normal visual acuity or corrected visual acuity. The participants provided written informed consent.

**Table 1:** Demographics and Clinical characteristics (*N* = 74)

	Suicide attempters	Non-suicide attempters	Healthy controls	Statistics F/ *χ*^2^	*p* value	Post Hoc
Demographics: mean(SD)						
Sample size	26	26	22			
Mean age	36.88 ± 15.37	39.42 ± 11.21	43.36 ± 14.07	1.35	0.161	
Mean years of education	13.23 ± 3.13	12.08 ± 2.95	13.36 ± 3.13	1.34	0.227	
Sex(male/female)	8/18	12 /14	6 /16	2.20	0.33	
Diagnosis (MDD/BD/SCH)	16/6/4	12/8/6	–	1.26	0.53	
Clinical scales: mean (SD)						
Back’s Depression Inventory(BDI)	10.42 ± 9.94	5.70 ± 6.23	3.00 ± 4.24	6.35	0.003**	SA > NSA > HC
Beck’s Scale For Suicide Ideation(BSSI)	26.00 ± 18.67	6.23 ± 9.04	1.27 ± 3.39	27.31	0.000**	SA > NSA > HC
State Trait Anxiety Inventory(STAI)	47.23 ± 15.16	46.88 ± 10.47	38.59 ± 11.37	3.52	0.035*	SA,NSA > HC

Data are presented as mean (SD).* *P* < 0.05 ，** *P* < 0.01

*Abbreviations: MDD* major depressive disorder, *SCH* schizophrenia, *BD* bipolar disorder

### Ethics

The study has been reviewed and approved in accordance with the relevant guidelines and regulations. The study was approved by the Ethical Committee of Tianjin University (TJUE-2021-165) and the Ethics Committee of Tianjin Anding Hospital. Each patient provided written informed consent prior to enrolling in the study.

#### Ultimatum game (UG)

The UG is a socioeconomic decision-making game that involves two players agreeing on a split of 100 yuan (about $15.10). The proposer must offer a share to the responder. Then, the responder decides whether or not to accept the offer. If accepted, the money is shared according to the proposition; if refused, neither of the players gain benefit. Using the latest version of the UG, each participant played the role of responder to decide whether to accept or reject the five allocation schemes (i.e., 50/50, 60/40, 70/30, 80/20, and 90/10). 50/50 and 60/40 were considered fair offers, whereas 80/20 and 90/10 were considered unfair offers. In total, 100 trials were distributed over the five schemes (i.e., 20 trials for each distribution scheme). Before the experiment, the subjects were informed in advance that they would play the UG together with a anonymous partner. However, in reality, no one else was playing the game, and participants received no further information about the identities of the supposed players. All offers were actually assigned by the computer randomly and the order of trials were different from one participant to another. The participants were also told to play the UG and attempt to maximize their gains. They were instructed about the outcomes of accepting and rejecting the offers. After the experiment, the participants received gifts of a value corresponding to the total amount obtained in the experiment. According to their self-report, all the participants believed that they were interacting with human proposers during the task.

#### Experimental procedures

**Fig. 1:**
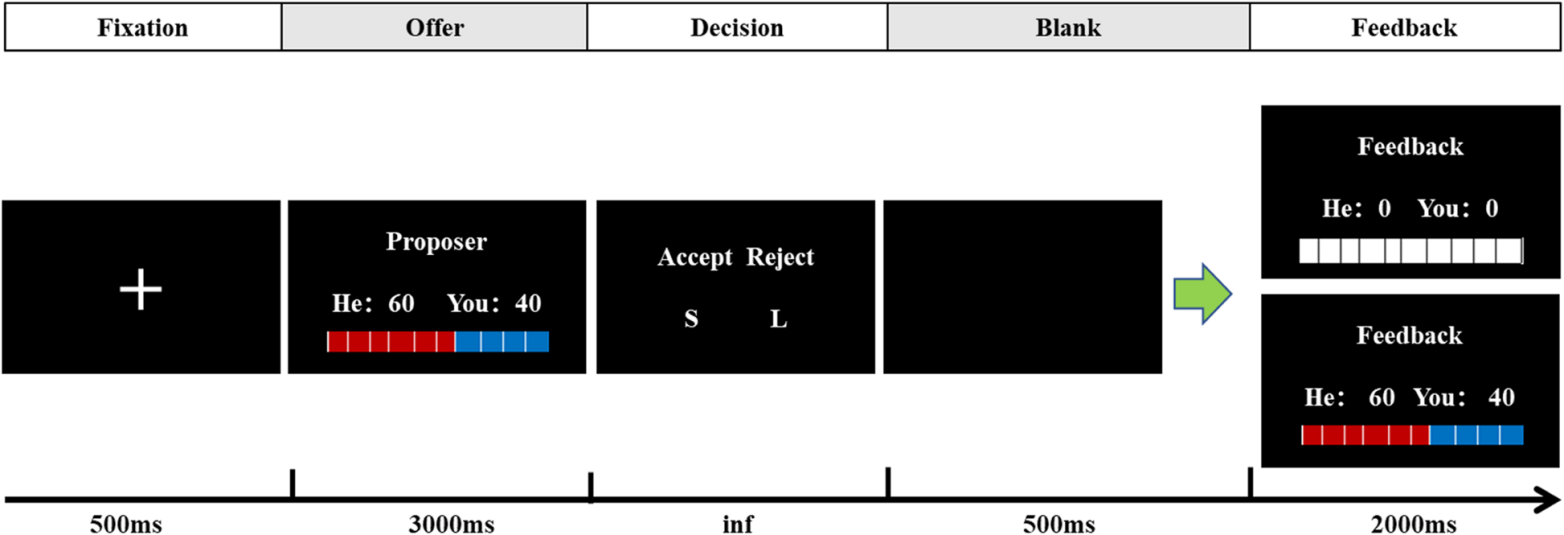
Illustration of the Ultimatum Game. In the task, each trial began with the instruction to press the spacebar. As soon as participants did, they were instructed to maintain their gaze on a central fixation cross for 500 ms. Next, the participants saw a message indicating an offer, and the participants can consider accepting or rejecting the offer. This time point was considered as our t = 0 for the ERP analyses and is marked with a horizontal black line. The response to the offer was displayed simultaneously when the participants pressed the button(S or L, representing acceptance or rejection of the offer by the responder, respectively) indicating their decision

The subjects first practiced for five trials before the formal experiment was performed for 100 trials. In each trial, we first presented the fixation point “+” for 500 ms, and the other party put forward the current round of allocation scheme for 3000 ms. Then, the subjects needed to make a key response, i.e., “accept” or “reject.” A blank screen was displayed for 500 ms, followed by the current round of allocation result for 2000 ms (Fig. 1). After the trials, the total amount obtained by the subjects was displayed on the screen. The overall experiment lasted for almost 90 min.

#### Psychophysiological recordings

After recruitment and consent, the participants were seated in an electrically shielded and light-attenuated room. The SynAmps2 EEG system was used to record the EEG. EEG datasets were recorded using 64 Ag/AgCl electrodes that were positioned in compliance with the 10/20 international electrode placement system. The predefined online parameters of data recording were 1000 Hz sampling rate and 0.01–70 Hz online bandpass filtering. The M2 electrodes served as the reference. For all electrodes, the impedance was kept below 5 kΩ during EEG recording.

### Data processing and analysis

#### ERP analysis

To conduct offline EEG analyses, we used MATLAB 2016a and the ERPLAB toolbox. EEG signals were first re-referenced to bilateral mastoid and down-sampled to 200 Hz, followed by down-sampling to 0.1–30 Hz, using an FIR filter, and partitioned into trials aligned at stimulus onset. We aligned all trials to a common baseline for each channel by subtracting the average of the 200-ms pre-stimulus interval from each trial’s waveform. The epochs were discarded if the voltage varied by more than 100 μV. Approximately 30 trials remained in each condition (fair: 50/50, 60/40; unfair: 80/20, 90/10). Finally, the mean waveforms were computed for each condition and participant, and these waveforms were used for further analyses.

Time-domain averaging was used to characterize N1, P2, and FRN for a cluster of six electrodes (FPz, Fz, FCz, Cz, CPz, and Pz) [53, 54]. For each condition, N1 was scored as the mean amplitude in the time window of 60–120 ms, whereas P200 and FRN were scored as the mean amplitudes in the time windows of 180–220 and 250–350 ms post-stimulus intervals, respectively.

#### PAC analysis

**Fig. 2:**
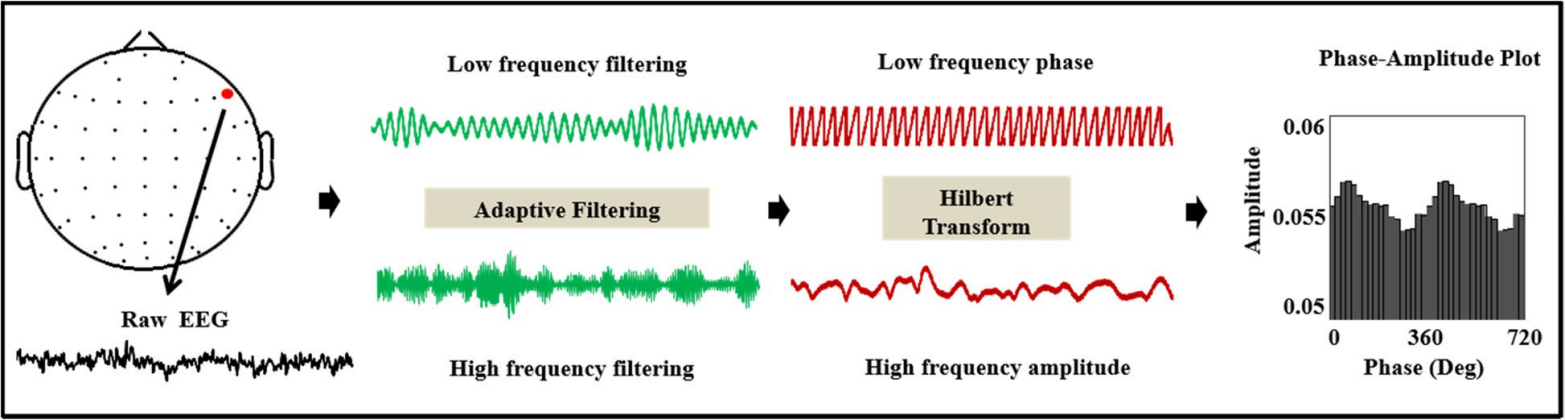
The calculation processes of the Modulation Index in a 3 s data file. From the raw signal shown in black, low- and high- frequency time series components denoted in green, respectively, were extracted using the finite impulse response filters. For both signals, the low-frequency phase and high-frequency envelope were calculated using the Hilbert transform highlighted in red. By binning the envelope values for each phase bin, the high-frequency amplitude distribution (or amplitude probability) for a phase was calculated

Because of its role in information processing, impaired cognitive function in humans is related to theta-gamma and beta-gamma coupling [55, 56]. To determine whether theta-gamma and beta-gamma coupling in SAs are affected, we measure the PAC from frontal, central, temporal, parietal, and occipital brain regions in the three groups. PAC was calculated using the Kullback-Leibler–based modulation index (MI) method, which has been described previously [57] (see in Fig. 2). In brief, the EEG signal from each channel was filtered separately to extract the phase and amplitude at specified frequencies, using a 2-way FIR1 filter (eegfilt.m with fir1 parameters). To extract the phase, we filtered all frequencies at 4–28 Hz (with a 2-Hz bandwidth) individually and extracted the phase from this signal using Hilbert transform. Similarly, for the amplitude component of the signal, we filtered all frequencies at 50–140 Hz (using a 10-Hz bandwidth) and extracted the amplitude from the filtered signal using Hilbert transform. For each frequency pair, the distribution of the instantaneous amplitude envelope was computed for every 20° interval during the instantaneous phase, thereby creating 18 phase bins. The mean amplitude for each bin was normalized to the sum of the mean amplitude for all bins, thereby creating a distribution similar to the probability distribution. The probability distribution was compared to a uniform distribution using Kullback–Leibler distance measure to derive the coupling (MI). The MI for each frequency pair could then be displayed as a comodulogram. For PAC calculations, the 3-s data file (defined as − 1000 to 2000 ms relative to the visual stimulus onset, followed by selecting correctly encoded trials) was analyzed, except for segments containing artifacts. There was no significant difference in the epochs of data included in the final analysis between groups.

#### Statistical analysis

The data were analyzed using SPSS (version 26.0; IBM Corp., Armonk, NY, USA). We used a repeated-measures analysis of variance (ANOVA) with groups (SA, NSA, and HC) as between-subjects factor and condition (fair and unfair) as within-subjects factor to detect significant changes in ERP data. A non-parametric Kruskal–Wallis test was used to analyze the PAC data. We conducted Bonferroni’s correction to correct for multiple comparisons. Spearman’s correlation and logistic regression analyses were used to identify risk factors associated with suicide attempts. Differences were considered to be statistically significant when *p*-values were < 0.05.

## Results

### Demographics

Table 1 presents the demographic data of participants. As indicated by ANOVA, there was no significant difference in age between the three groups. The current diagnosis was recorded as MDD/BD/SCH. The Chi-square test showed no significant differences in gender or current diagnosis among the three groups. The results showed significant differences in terms of BDI, BSSI, and STAI scores between the three groups. Post hoc testing revealed that BDI and BSSI scores were significantly higher for the SAs than for NSAs and HCs. The STAI scores were higher for the SAs and NSAs than for HCs.

### Electrophysiological data

#### ERPs

**Fig. 3:**
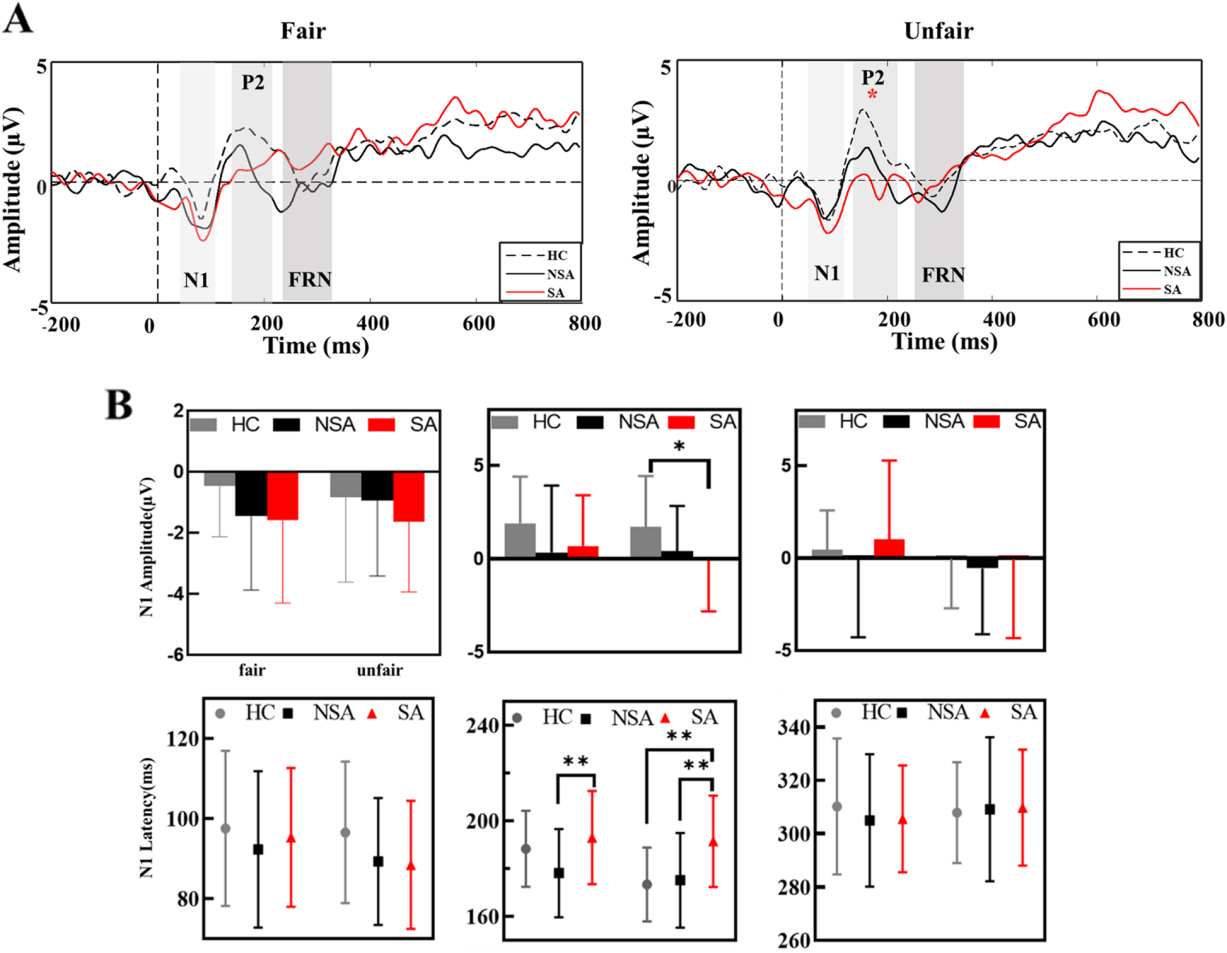
Grand average waveforms. (**a**) Grand average waveform for the electrode average of six electrodes (FPz, Fz, FCz, Cz,CPz, and Pz) for three groups, following fair conditions (50/50; 60/40) and unfair conditions (80/20; 90/10). (**b**) In fair and unfair conditions, the amplitude and latency of N1, P2, and FRN in there groups were measured. * *p* < 0.05

Figure 3A depicts the grand-average ERP waveforms for SAs (red line), NSAs (black line), and HCs (black dotted line) in each condition at the six (FPz, Fz, FCz, Cz, CPz, and Pz) electrodes.

#### N1

SAs showed a slight increase in N1 amplitude compared to NSA and HC groups (Fig. 3A). However, no group effects or interaction effects were observed for N1 amplitude and latency (Fig. 3A and B).

#### P2

The main effect of group was significant [F = 3.20, *p* = 0.04, η_*p*_2 = 0.094], and post hoc analyses indicated that SAs exhibited smaller P2 amplitude than HCs. Importantly, a significant interaction effect was observed between condition and group. A simple effects analysis showed that the group effect was only significant for unfair condition, indicating a smaller P2 amplitude in the SA group in response to unfair offers compared to HCs [F = 3.26, *p* = 0.04, η_*p*_^2^ = 0.094]. Meanwhile, for P2 latency, there was a significant main effect of group [F = 5.65, *p* = 0.005, η_*p*_^2^ = 0.148], and post hoc analyses indicated a greater prolonged P2 latency for SAs compared to NSAs (*p* = 0.027) and HC (*p* = 0.002) groups. Moreover, a significant interaction effect was observed between group and condition [F = 4.86, *p* = 0.01, η_*p*_^2^ = 0.130]. A simple effects analysis found that P2 latency was increased in the SA group compared to the NSA group in fair (*p* = 0.006) and unfair (*p* = 0.002) conditions, and was prolonged in the SA group compared to the HC group in unfair conditions (*p* = 0.003) (Fig. 3B). Furthermore, Spearman’s bivariate correlation analysis showed a negative correlation between P2 latency and BSSI in the SA group (*r* = 0.55, *p* = 0.01, Table 2).

**Table 2:** Descriptive statistics and correlations insuicide attempters

Measure	M (SD)	BDI	STAI	BSSI
1 F(TGC)	0.00191 (0.00010)	r = 0.06 *p* = 0.77	r = 0.09 *p* = 0.65	r = 0.34 *p* = 0.09
2 C(TGC)	0.00193 (0.00013)	r = 0.10 *p* = 0.62	r = 0.14 *p* = 0.49	r = 0.38 *p* = 0.05
3 T(TGC)	0.00194 (0.00014)	r = 0.17 *p* = 0.42	r = 0.12 *p* = 0.56	r = 0.29 *p* = 0.15
4 u_F(TGC)	0.00198 (0.00018)	r = − 0.37 *p* = 0.06	r = − 0.39 p = 0.05	r = − 0.2 *p* = 0.20
5 u_C(TGC)	0.00194 (0.00019)	r = − 0.20 *p* = 0.33	r = − 0.26 *p* = 0.21	r = − 0.17 r = 0.42
6 u_T(TGC)	0.00199 (0.00019)	r = − 0.41 p = 0.04*	r = − 0.46 p = 0.02*	r = − 0.33 *p* = 0.10
7 C(BGC)	0.00050 (0.00007)	r = 0.10 p = 0.62	r = 0.14 p = 0.49	r = 0.38 p = 0.05
8 T(BGC)	0.00052 (0.00006)	r = 0.17 p = 0.42	r = 0.12 p = 0.56	r = 0.29 p = 0.15
9 u_C(BGC)	0.0005 (0.00007)	r = − 0.22 *p* = 0.29	r = − 0.27 *p* = 0.19	r = − 0.09 *p* = 0.68
10 u_T(BGC)	0.0005 (0.00007)	r = − 0.25 *p* = 0.22	r = − 0.28 *p* = 0.16	r = − 0.13 *p* = 0.53
11 u_ P2 amplitude	- 0.098 (2.72)	r = 0.22 *p* = 0.31	r = 0.18 *p* = 0.40	r = 0.11 p = 0.62
12 u_ P2 latency	191.42 (19.14)	r = 0.38 *p* = 0.07	r = 0.45 *p* = 0.03*	r = 0.55 *p* = 0.01*

*Abbreviations: STAI* State Trait Anxiety Inventory, *BDI* Beck’s Depression Inventory, *BSSI* Beck’s Scale For Suicide Ideation, *F* Frontal, *T* Temporal, *TGC* the Coupling value of MI for theta phase and gamma amplitude, *BGC* Coupling value of MI for beta phase and gamma amplitude, *SD* standard deviation. ** *p* < 0.01. * *p* < 0.05

#### FRN

**Fig. 4:**
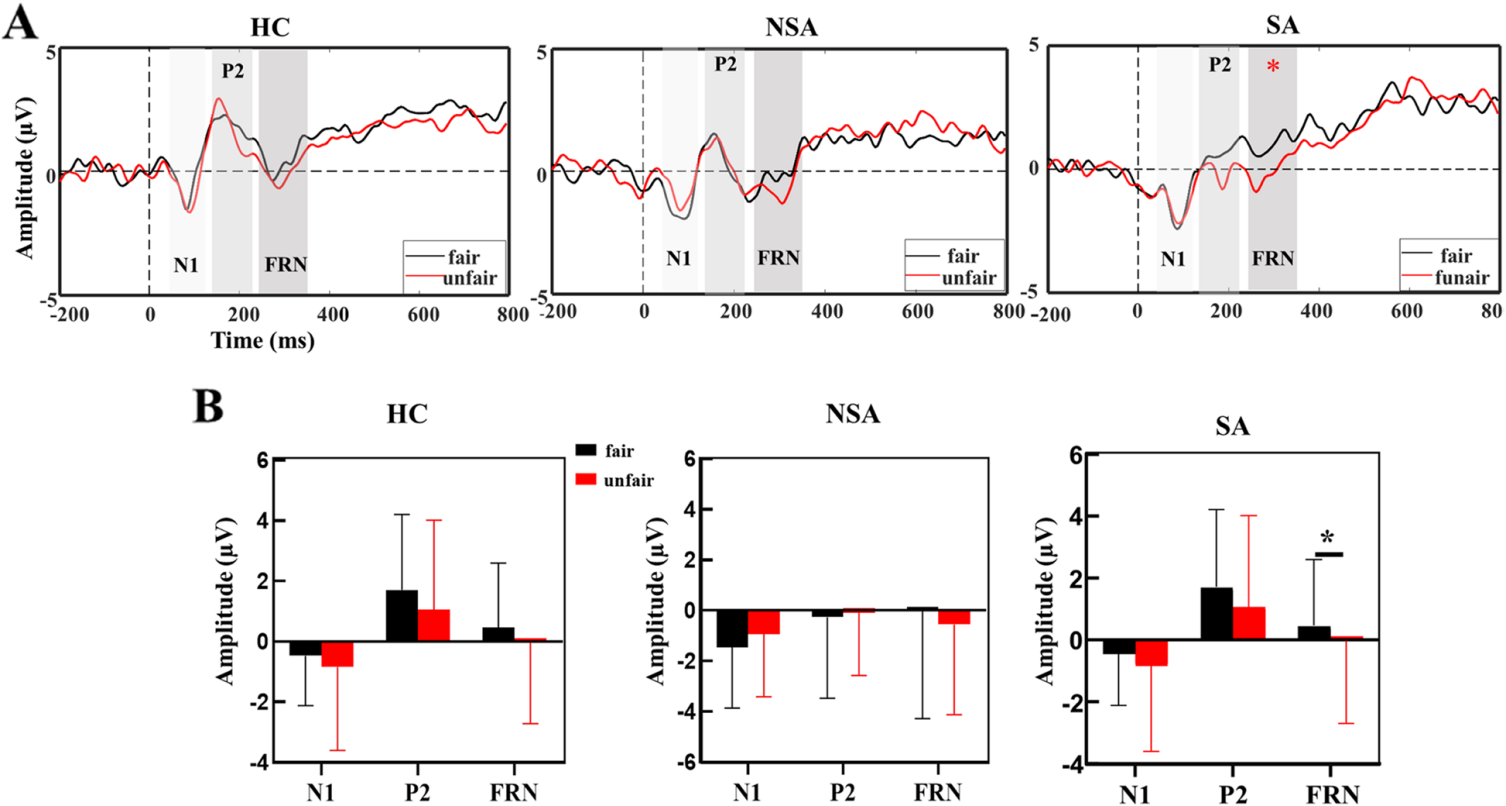
Grand-average ERP waveforms. (**a**) ERP difference of grand-average waveform for the electrode average of six electrodes (FPz, Fz, FCz, Cz, CPz, and Pz) between fair proposals (50/50; 60/40) and unfair proposals (80/20; 90/10) in each groups. (**b**) Amplitude of N1, P2, and FRN in the two conditions were measured in each group. * *p* < 0.05

As can be seen, the main effects of group and interaction did not reach significance for FRN components. However, there was a marginally significant condition effect [F = 3.10, *p* = 0.08, η_*p*_^2^ = 0.048], indicating that FRN was more negative in response to unfair offers than fair offers. Post hoc analysis to analyze the difference in FRN between fair and unfair conditions among the three groups, revealing that SAs exhibited a significant increase in FRN for the unfair condition (*p* = 0.015), whereas such a trend was absent in the NSA and HC groups. The difference in FRN between fair and unfair conditions in the three groups is shown in Fig. 4A and Fig. 4B.

#### Pac

To further explore decision-making, we calculated the MI to assess the PAC differences among the three groups while responding to fair and unfair offers in five brain areas (frontal, central, temporal, parietal, and occipital).

**Fig. 5:**
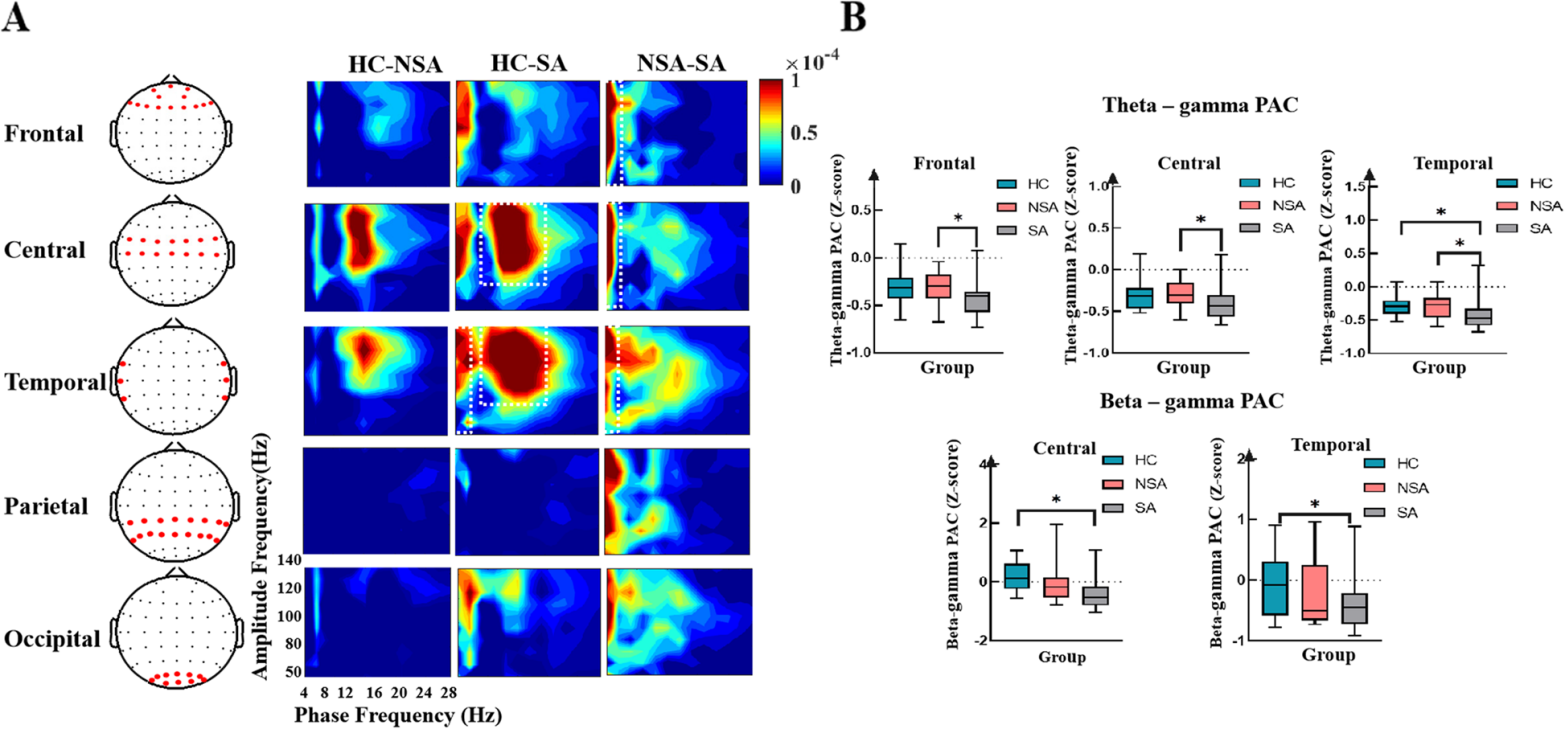
(**a**) Group comodulograms showing in the fair condition the difference in modulation index across three groups in five brain regions. (**b**) The top boxplots show MI values averaged (converted to Z scores) over theta (4–8 Hz) phase and broadband gamma (50–140 Hz) amplitude. The bottom boxplots show MI values averaged (converted to Z scores) over beta (13–28 Hz) phase and broadband gamma (50–140 Hz) amplitude. Black asterisks represent significant comparisons (*p < 0.05, **p < 0.01)

As can be seen in Fig. 5A and B, in the fair condition, the SA group exhibited deficits in theta-gamma PAC in frontal (*p* = 0.013), central (*p* = 0.019), and temporal (*p* = 0.016) regions compared to the NSA group. When compared to HCs, theta-gamma PAC (*p* = 0.016) was significantly decreased in the temporal region, and beta-gamma PAC was significantly reduced in the central (*p* = 0.001) and temporal (*p* = 0.009) regions in the SA group. However, there were no significant differences between the NSA and HC groups.

**Fig. 6:**
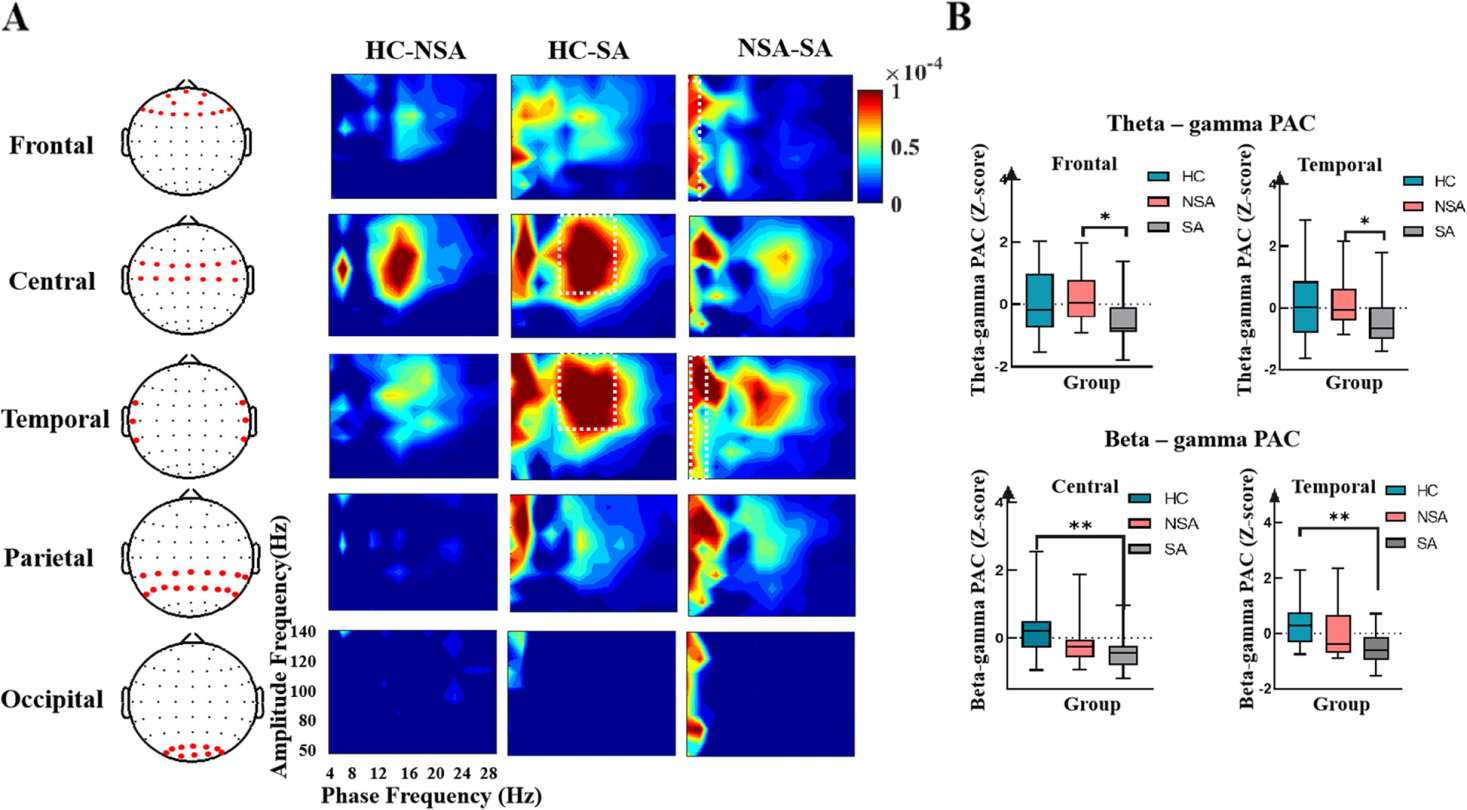
(**a**) Group comodulograms showing in the unfair condition the difference in modulation index across the three groups in five brain regions. (**b**) The top boxplots showing MI values averaged (converted to Z scores) over theta (4–8 Hz) phase and broadband gamma (50–140 Hz) amplitude. The bottom boxplots show MI values averaged (converted to Z scores) over beta (13–28 Hz) phase and broadband gamma (50–140 Hz) amplitude. Black asterisks represent significant comparisons (*p < 0.05, **p < 0.01)

Figure 6A and B shows the inter-group differences in five brain regions when responding to unfair offers. Compared to the NSA group, the SA group exhibited deficits in theta-gamma PAC in the frontal (*p* = 0.013) and central (*p* = 0.044) regions. Furthermore, beta-gamma PAC was significantly attenuated in the central (*p* = 0.045) and temporal (*p* = 0.001) regions in the SA group compared to HCs. Spearman’s bivariate correlation analysis found a negative correlation between theta-gamma PAC in the temporal region and the BDI scores (r = − 0.41, *p* = 0.04), the STAI scores (r = − 0.46, *p* = 0.02).

### Logistic regression model

**Fig. 7:**
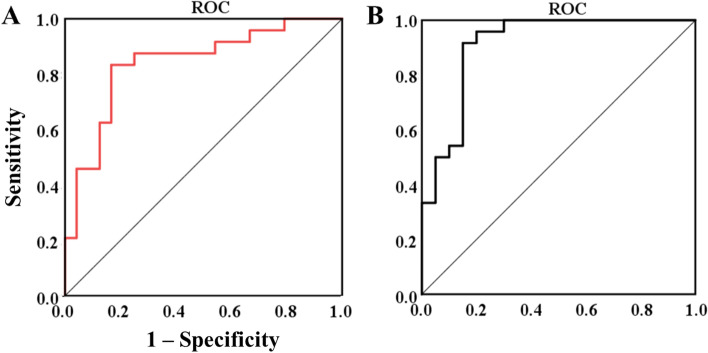
(**a**). Receiver operating characteristic (ROC) curve analysis graph (AUC = 0.84). (**b**). ROC curve analysis graph (AUC = 0.91)

The statistically significant variables were used in logistic regression analysis. The logistic regression model was developed with suicide attempt status as the dependent variable. We selected theta-gamma PAC, beta-gamma PAC, and P2 latency as independent variables. Classification logic regression was used to evaluate the indicators, and the results showed that the model was statistically significant (*p* < 0.05). The area under the receiver operating characteristic curve (AUC) in the logistic regression model was 0.84 for the SA/NSA groups (see Fig. 7A) and 0.91 for the SA/HC groups (see Fig. 7B). These results indicated that theta-gamma PAC, beta-gamma PAC, and P2 latencywere the most important risk factors for suicide attempt.

## Discussion

The present study explored the electrophysiological bases of decision-making and social interactions in suicide attempters during UG. We found that defective P2, theta-gamma PAC, and beta-gamma PAC are the pathophysiology in SA during the early processes of social decision-making. P2 was sensitive to motivational information of stimuli, and changes in P2 may be a marker of social cognition, which is reduced during unfair decision-making. The evidence for PAC loss suggests a common mechanism for social decision-making impairment. However, we did not find a different N1 in the SA group. The unfair offers elicited greater negative-going FRN than fair offers in the SA group, which exposed abnormal sensitivity to unfairness. This study contributes to research on the electrophysiological bases of decision-making in suicide attempters and have potential clinical implications.

Electrophysiologically, three ERP indicators (N1, P2, and FRN) were extracted to reveal group differences in decision-making processing. We did not find a difference in N1 composition in the SA group, which is consistent with previous studies that showed that there was no difference in early perceptual processing during decision-making tasks between patients with high and low symptom severity [58]. The results of P2 suggested that SAs exhibited automatic abnormal processing to unfairness in the early processing stage of decision-making. Globally, the P2 is related to higher-order processes that involve the comparison of the eliciting event of the ERP with internal representation. The diminished amplitude of the P2 in the UG is due to a lower engagement of attention. It has been established that the attention level is related to mental effort [19], and we previously reported that a reduced allocation of attention results in a reduced P2 amplitude [59]. Furthermore, the P2 amplitude decreased in both SA and NSA, and cannot rule out the influence of mental disorders on the results. The later stage of fairness processing is represented by FRN, a critical ERP component originating near the anterior cingulate cortex, which was related to the emotional motivation significance of the results [60, 61]. The significant difference found between fair and unfair conditions further supports the unique electrophysiological differences when dealing with information which is contrary to the expected results. The enhanced FRN in unfair condition indicated that SA were based on fairness perception rather than on personal interests during UG, which may lead to difficulties in social communication. In this study, unfair offers elicited greater negative-going FRN than fair offers in the SA group, implying that individuals considered unfair offers to be an unfavorable outcome that violated social norms. The unfair offers represented lower benefit and equality, which were worse than their expected ones, and thus elicited a larger FRN in the SA group. In contrast, the NSA and HC groups mainly focused on their personal interests in UG and placed less value on the fairness embedded in the offers. This tendency distinguished the SA group from the NSA and HC groups, whose punishment decisions were sensitive to personal cost. The initial cost of our study was 100 ¥, which may be perceived as a high initial cost by Chinese participants. This explains why no difference in FRN was found between fair and unfair conditions between the HC and NSA groups. Suicide attempters’ relative insensitivity to the cost of retaliation may lead to uncompromising, catastrophic responses to conflict.

We evaluated the abnormal neuronal oscillations in suicide attempters to explore the neural mechanism underlying decision-making deficits. We observed that SAs showed significant changes in θ-γ PAC in the central and temporal lobes compared to the NSA group. Compared to HCs, the central and temporal lobes of suicide attempters showed deficits in β-γ PAC. Recent reports have related PAC to context-based rule retrieval and working memory [62, 63]. However, it remains unclear whether and how PAC is involved in decision-making. Our results confirmed the existence of PAC in decision-making and revealed coupling defects in the frontal, central, and temporal regions, which may explain the neuronal dysfunction in the decision-making task in the SA group. Our findings increased the potential application of PAC detection and regulation in the diagnosis and treatment of neurological diseases.

Using correlation analysis, we explored the biological and psychological bases of suicide attempters. Our results showed that greater P2 latency was positively correlated with BSSI and BDI scores, which may reflect the excessive sensitivity of suicide attempters to social events, resulting in abnormal reflection of early ERP components and leading to decision-making deficits. We also found that STAI and BDI scores were negatively correlated with θ-γPAC in the temporal lobe. This neural deficit may indicate that there is a lack of top-down processing in decision-making tasks, resulting in abnormal decision-making. Finally, our logistic regression analysis showed that incorporating these indicators into the model achieved good classification results. The results emphasized a significant role of neurobiological indicators in distinguishing between SA, NSA, and HC individuals. P2 latency, θ-γ PAC, and β-γ PAC may be biomarkers of suicide attempts during decision-making. These results may help us to better understand the role of decision-making response in suicide behavior, which is needed for further research.

Social decision-making in suicide is a neglected area. To the best of our knowledge, this is the first study to combine suicide-related neurophysiological indicators in decision-making and perform logistic regression analyses to differentiate the SA group from the NSA and HC groups. This paper mainly explored the neural responses to decision-making in suicide attempters and used the mental disorder patients who have not attempted suicide but did not exclude suicidal ideation to eliminate the impact of mental disorders on decision-making. Our previous studies have shown that abnormal brain neural activity may be a potential biomarker of cognitive impairment in suicide attempters [64]. Further research is needed to comprehensively explore the mechanisms of suicide behavior and to incorporate biological or psychological features that will enable the construction of a more precise classification model. Additionally, a recent Electrocorticography (EcoG) study showed that therapeutic deep brain stimulation (DBS) acutely reduced cortical PAC [65, 66], suggesting that this marker could be used for the physiology-based optimization of DBS settings in the clinic, or even allow continuous device adjustment in real time based on cortical activity, using a scalp contact for feedback control in closed-loop DBS. The transcranial altermating current stimulation (tACS) and transcranial direct current stimulation (tDCS) have been reported to improve individual cognitive ability, which worked by changing theta–gamma PAC. Thus, if PAC is disrupted in suicide attempters with mental disorder during social decision-making, then tACS or tDCS may be promising prospective treatment [67, 68].

The current study has several limitations. First, the usefulness of findings needs to be further validated, with preferably larger sample sizes. The clinical diagnosis of psychiatric disorders is not consistent. Additionally, the demographic data may have a certain impact on the differences among SA, NSA and HC groups in the study. Future studies should strictly control the experimental conditions, using a standardized approach exploring the social decision performance characteristics of suicide attempters in a larger and single sample of psychiatric disorders. Second, recalled information about past suicide behaviors and most severe past depressive episodes may not be complete despite using MINI and self report aseessment. This study recorded lifetime suicide attempts, rather than a recent suicide attempt. In addition, the patients we recruited were drug-dependent, and the influence of drugs has not been ruled out. The more conditions and restrictions were needed for research in the future. Furthermore, It will be interesting to consider the differences of electrophysiological biomarks between high and low suicide ideation and suicide attempts. Third, we foucsed on the unfairness stimulus evaluation in the early stage of decision-making, rather than the accept and reject decisions wich could really measure the process of decision-making, we will examine the mechanims of social decision-making in our later study. Finally, the research mainly used game tasks in the context of computer simulation. However, decisions are more complex in real situations. The real-time interactive feedback between both sides and the use of different strategies will affect the participants’ performance. Therefore, the interpretation and application of the present study results require caution.

## Conclusion

Our study explored the suicide-related neurophysiological indicators during UG task and performed logistic regression analyses to distinguish the SA group from the HC and NSA groups. Our previous work showed that the SA group exhibited abnormal fairness effect on ERP components relative to the NSA and HC groups. The PAC in the frontal, central, and temporal regions may explain the neuronal dysfunction in the decision-making task of the SA group and further confirmed the potential value of PAC in the diagnosis of brain diseases. The biomarkers developed in our work could be used as potential signals to bring new light to understand the neural mechanism of unfair decision-making in suicide attempters.

## Data Availability

The datasets generated and/or analyzed during the current study are not publicly available due to the used data protection declaration, but are available from the corresponding author on reasonable request.

## Abbreviations

SA: suicide attempters
NSA: non-suicide attempters
HC: healthy controls
PAC: phase-amplitude coupling
